# An evaluation of the impact of a multidisciplinary team, in a single centre, on treatment and survival in patients with inoperable non-small-cell lung cancer

**DOI:** 10.1038/sj.bjc.6602825

**Published:** 2005-10-18

**Authors:** L M Forrest, D C McMillan, C S McArdle, D J Dunlop

**Affiliations:** 1 University Department of Surgery, Royal Infirmary, Glasgow G31 2ER, UK; 2 Department of Medical Oncology, Royal Infirmary, Glasgow G31 2ER, UK

**Keywords:** non-small-cell lung cancer, radiotherapy, chemotherapy, multidisciplinary team, survival

## Abstract

Treatment and survival of patients with inoperable Non-small-cell lung cancer in 1997 (*n*=117) and 2001 (*n*=126), before and after the introduction of a multidisciplinary team, was examined in a single centre. There were no differences in age, sex and extent of deprivation between the two years. However, in 2001, 23% of patients received chemotherapy treatment compared with 7% in 1997 (*P*<0.001). Median survival in 2001 was 6.6 months compared with 3.2 months in 1997 (*P*<0.001).

Lung cancer is the commonest cause of cancer death in North America and Western Europe. For example, each year in the UK there are approximately 38 000 new cases and approximately 33 000 deaths (Cancerstats, 2003; www.cancerresearchuk.org).

Non-small-cell lung cancer (NSCLC) accounts for approximately three-quarters of all lung cancer cases. Survival remains poor. For example, in Scotland, the median survival for patients with NSCLC is approximately 5 months (Gregor *et al*, 2001). Therefore, it is important to ensure that the treatment of these patients is optimal and that active treatment is offered to all patients who may benefit (Gregor *et al*, 2001; Jack *et al*, 2003).

This rationale has driven the introduction of specialised care by multi-disciplinary teams to improve the delivery of care and treatment to patients with lung cancer (Selby *et al*, 1996; Alberts *et al*, 2003). However, to date, few studies have specifically examined the effect of the multidisciplinary team on the treatment and survival of patients with NSCLC.

The aim of the present study was to examine the impact of the introduction of a multidisciplinary team on survival of patients with inoperable NSCLC.

## PATIENTS

Patients diagnosed with inoperable NSCLC (stage III/IV) at the Royal Infirmary, Glasgow, prior to and following the introduction of a multidisciplinary team in 1998 were included in the study. The multi-disciplinary team consisted of two respiratory physicians, two surgeons, a medical oncologist, a clinical oncologist, a palliative care physician, a radiologist and a specialist respiratory nurse.

Information was abstracted from the case notes by a specially trained research nurse (LMF). Data for the year 1997 prior to the introduction of a multidisciplinary team were collected retrospectively and those for 2001 were collected prospectively. Patient details, including age, sex, extent of deprivation, tumour type, stage and type of treatment were obtained from the patient's records.

The extent of deprivation was defined using the Carstairs Index, an area-based measure derived from the 1991 census data based on the postcode of residence at diagnosis. (Carstairs and Morris, 1991).

Patients were staged in accordance with the American Thoracic Society TNM classification on the basis of clinical findings, chest X-ray, and, where appropriate, bronchoscopy, liver ultrasound, isotope bone scan and computerised tomography of the thorax (Mountain, 1991).

Patients were considered to have undergone active treatment if they received chemotherapy (mainly cisplatin based) and/or radical radiotherapy. Patients receiving palliative radiotherapy and/or palliative care (symptom control) were considered to have had palliative treatment.

The study was approved by the Research Ethics Committee of the Royal Infirmary, Glasgow.

### Statistics

Comparisons between groups of patients (1997 and 2001) were carried out using contingency table analysis (*χ*^2^) as appropriate. Univariate survival analysis was performed using the Kaplan–Meier method with the logrank test. Deaths up to 30 September 2004 were included in the analysis. Analysis was performed using SPSS software (SPSS Inc., Chicago, IL, USA).

## RESULTS

Of 323 (167 patients in 1997 and 156 patients in 2001) patients with histological or cytological evidence of NSCLC, 243 (117 patients in 1997 and 126 patients in 2001) patients underwent formal staging (Fisher's exact test, *P*=0.035). The characteristics of patients (*n*=243) with inoperable NSCLC (stage III/IV) treated in 1997 and 2001 are shown in Table 1.

Overall, the majority of patients were over 60 years of age, male, deprived, and had stage IV disease. There were no differences in age, sex and extent of deprivation between the two years. However, there was a decrease in the proportion of patients presenting with stage IIIa disease in 2001 (*P*=0.015).

In 2001, 23% of patients received chemotherapy treatment compared with 7% of patients in 1997 (*P*<0.001). In 2001, 44% of patients received palliative care only compared with 58% of patients in 1997 (*P*=0.045). On follow-up, 116 of the 117 patients diagnosed in 1997 had died compared with 116 of 126 patients diagnosed in 2001 (Fisher's exact test, *P*=0.011). Median survival was significantly higher in patients treated in 2001 compared with those treated in 1997 (6.6 *vs* 3.2 months, *P*<0.001).

## DISCUSSION

The present study, to our knowledge, is the first to examine the impact of the introduction of a multidisciplinary team on the survival of patients with inoperable NSCLC presenting to a single centre.

In the present study, the introduction of a multidisciplinary team was associated with a change in the treatment of patients with inoperable NSCLC. In particular, more patients received chemotherapy and fewer patients received palliative care only. Although the proportion of patients who received chemotherapy following the introduction of the multidisciplinary team appears low, more than half of the patients studied are from an area of multiple deprivation. This is not surprising, since eight of the 15 most deprived areas in the UK are in Glasgow (Gordon *et al*, 1999).

A possible explanation of the results of the present study is that indications for chemotherapy in the earlier period (1997) may not have been as strong as in the latter period (2001). However, in 1997, the use of chemotherapy in this centre was primarily determined by a medical oncologist (DJD) according to referral, indication and clinical need. At this time, there was good evidence of the benefit of chemotherapy in patients with inoperable NSCLC (Non-Small Cell Lung Cancer Collaborative Group, 1995).

A significant problem in studies of this type is the phenomenon of ‘stage drift’, whereby in the latter study period patients are diagnosed at an earlier point in their disease course and so appear to have an improved outcome. In the present study, we chose to study only those patients with a clinical stage defined as inoperable in both time periods in an attempt to minimise this problem. Consistent with this approach was that similar numbers of patients were studied and that age, sex and deprivation were similar in the two time periods, independent of stage.

The introduction of a multidisciplinary team was associated with an increase in the proportion of patients being staged, presenting with stage IIIb disease and receiving chemotherapy. This was associated with a doubling of survival in patients with stage III disease. Although there was evidence of ‘stage drift’, it was towards more advanced disease and therefore would not account for the improvement in survival. Furthermore, the improvement in the survival of the cohort as a whole would suggest that, in the present study, treatment improved survival. Therefore, the results of the present study appear to confirm the value of a multidisciplinary approach to the management of patients with inoperable NSCLC.

## Figures and Tables

**Table 1:** Baseline characteristics of patients (*n*=243) with inoperable NSCLC (stage III/IV) in 1997 and 2001

	**1997**	**2001**	
	**Patients 117 (100%)**	**Patients 126 (100%)**	***P*-value**
*Age*			
<60 years	21 (18)	23 (18)	
>60 years	96 (82)	103 (82)	0.951
			
*Sex*			
Male	77 (66)	75 (60)	
Female	40 (34)	51 (40)	0.312
			
*Extent of deprivation*			
Affluent	1 (1)	7 (6)	
Intermediate	44 (38)	46 (36)	
Deprived	72 (61)	73 (58)	0.425
			
*Stage*			
IIIa	20 (17)	8 (6)	
IIIb	14 (12)	27 (22)	
IV	83 (71)	91 (72)	0.010
			
*Treatment*			
Radical radiotherapy	6 (5)	3 (2)	
Chemotherapy	8 (7)	29 (23)	
Palliative radiotherapy	35 (30)	38 (30)	
Palliative care only	68 (58)	56 (44)	0.003
			
Median survival (months)	3.2 (2.4–4.1)	6.6 (3.7–9.5)	<0.001

NSCLC=non-small-cell lung cancer.
